# Supplementary figures and images for: SIRT5 is a proviral factor that interacts with SARS-CoV-2 Nsp14 protein
Source: PLoS Pathog. 2022 Sep 12;18(9):e1010811. doi: 10.1371/journal.ppat.1010811 (PMC9499238; doi:10.1371/journal.ppat.1010811)

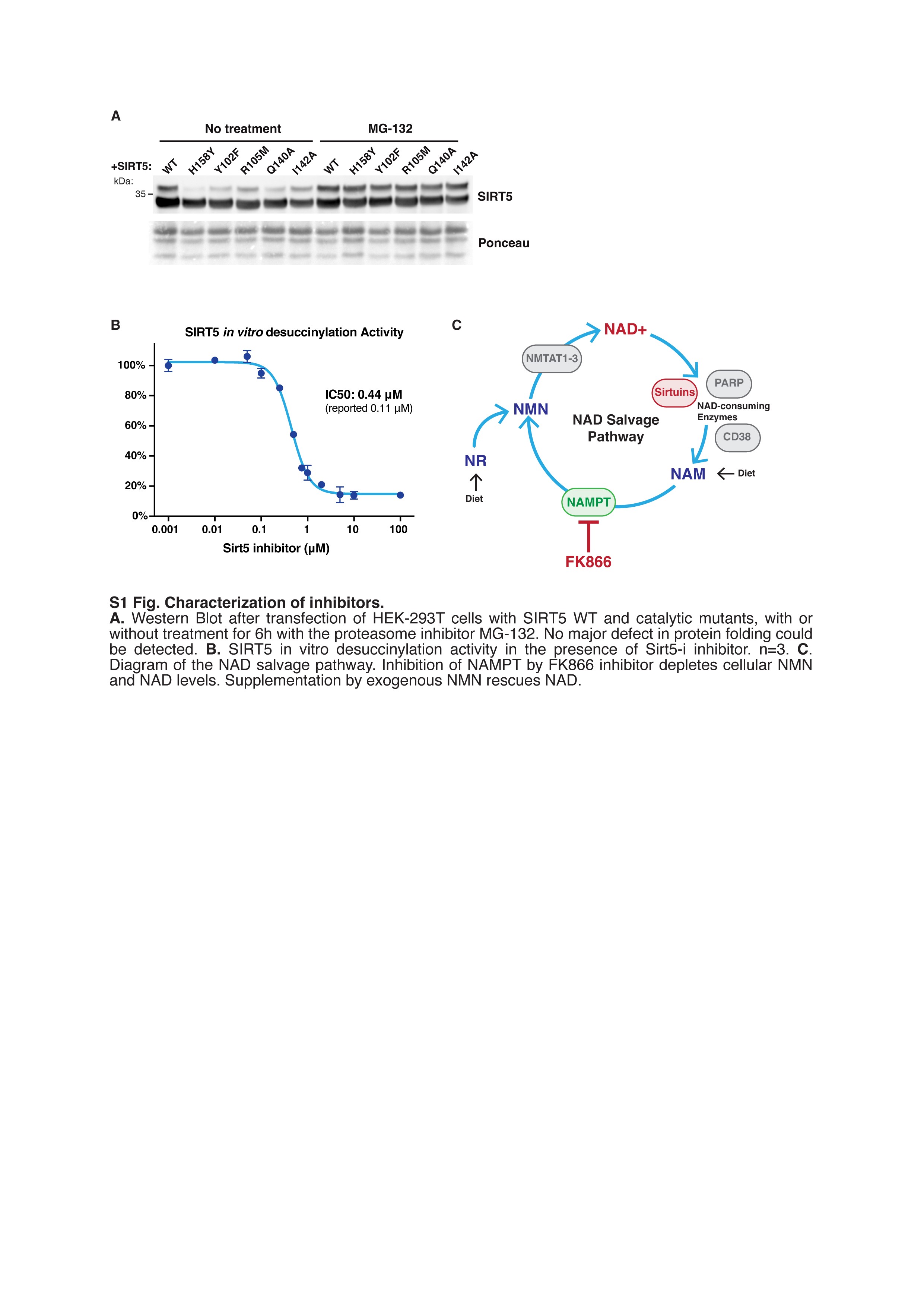

Supplement: S1 Fig — A. Western Blot after transfection of HEK-293T cells with SIRT5 WT and catalytic mutants, with or without treatment for 6h with the proteasome inhibitor MG-132. No major defect in protein folding could be detected. B. SIRT5 in vitro desuccinylation activity in the presence of Sirt5-i inhibitor. n = 3. C. Diagram of the NAD salvage pathway. Inhibition of NAMPT by FK866 inhibitor depletes cellular NMN and NAD levels. Supplementation by exogenous NMN rescues NAD. (TIF) [file ppat.1010811.s001.tif]

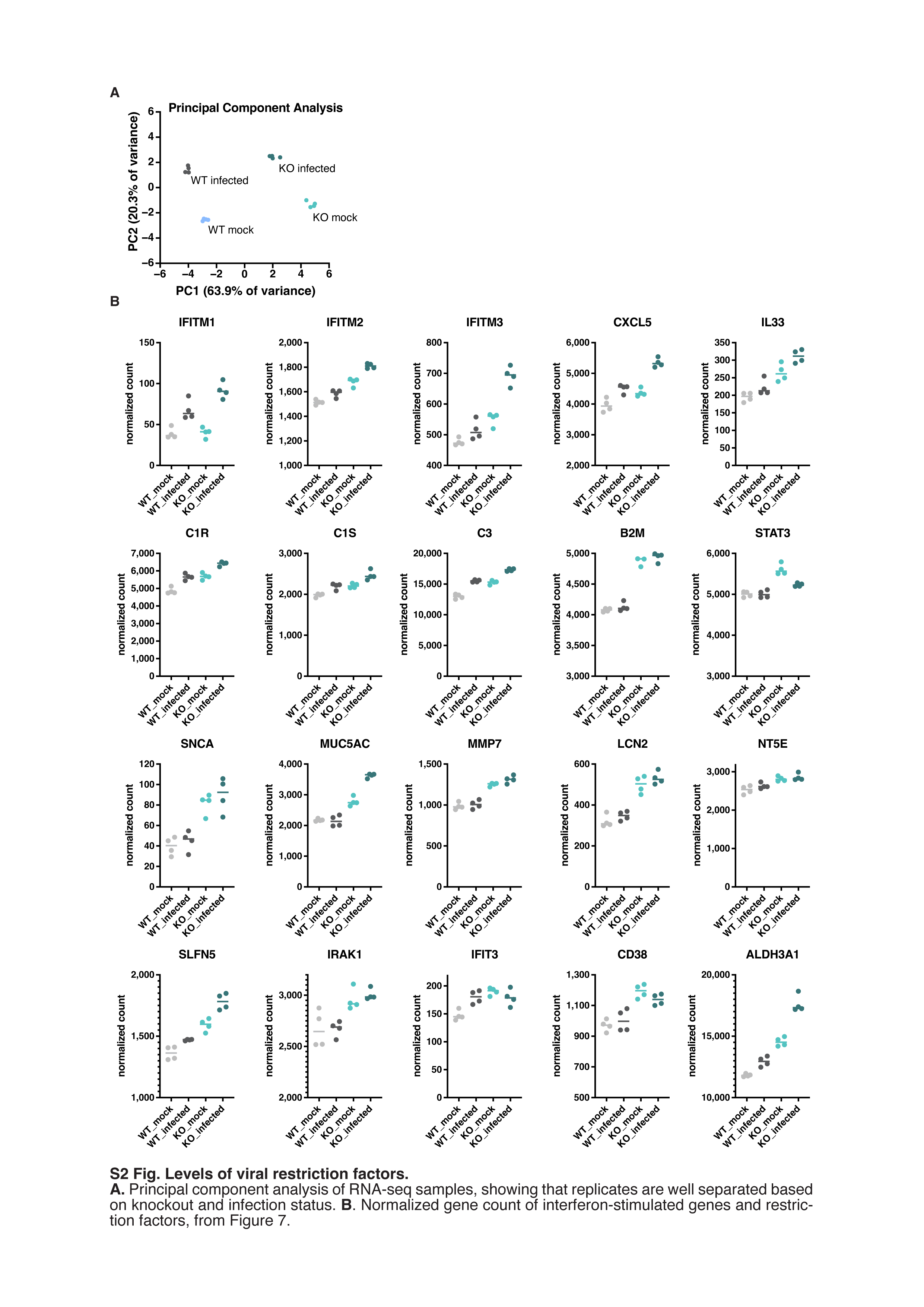

Supplement: S2 Fig — A. Principal component analysis of RNA-seq samples, showing that replicates are well separated based on knockout and infection status. B. Normalized gene count of interferon-stimulated genes and restriction factors, from Fig 7. (TIF) [file ppat.1010811.s002.tif]

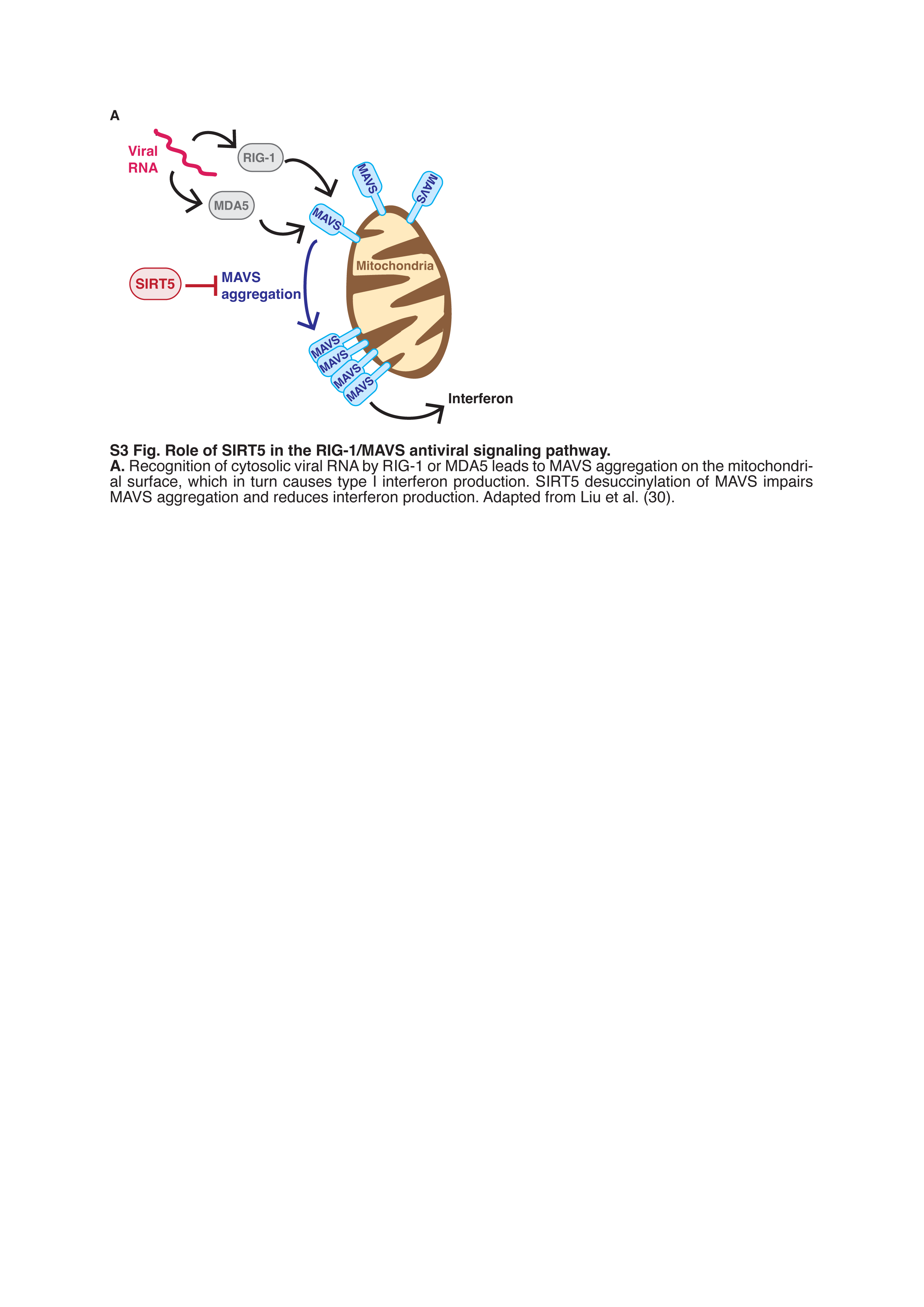

Supplement: S3 Fig — A. Recognition of cytosolic viral RNA by RIG-1 or MDA5 leads to MAVS aggregation on the mitochondrial surface, which in turn causes type I interferon production. SIRT5 desuccinylation of MAVS impairs MAVS aggregation and reduces interferon production. Adapted from Liu et al. [30]. (TIF) [file ppat.1010811.s003.tif]
