# Supplementary material for: SIRT5 is a proviral factor that interacts with SARS-CoV-2 Nsp14 protein
Source: PLoS Pathog. 2022 Sep 12;18(9):e1010811. doi: 10.1371/journal.ppat.1010811 (PMC9499238; doi:10.1371/journal.ppat.1010811)
Supplement: S3 Data — CRISPR gRNA sequences, primer sequences, antibodies, and small molecule inhibitors used in the study. (PDF) [file ppat.1010811.s006.pdf]

S1 Table - Reagents

CRISPR gRNAs (Knockout in A549-ACE2)

	gRNA 1	gRNA 2	gRNA 3
SIRT1	**CAAAGGAUAAUUCAGUGUCA**	**AUAGCCUUGUCAGAUAAGGA**	**UUGAUACAGGAAAUAUAUCC**
SIRT5	**GUGCAGCUCAUCGAUGUUCU**	**AGCCGAGUGUGAGACCCGGC**	**CCGGGACGGGUUGUGGGCAA**
MAVS	**GCTGGTAGCTCTGGTAGACAG**		

CRISPR genotyping primers (*SIRT1-SIRT5* Knockout in A549-ACE2)

	Forward	Reverse	Sequencing
SIRT1	**GCATATGACAGCAACCGTCC**	**GCTTTATCTCCACTTCTCGATG G**	**GTGTCGCATCCATCTAGATAC TTTAAAAT**
SIRT5	**GCATCTGCCATGTTGTTTGA**	**CTGAAACAGCAGGACAGGTG**	**CATCTGCCATGTTGTTTGAAC ATAGT**

CRISPRi gRNAs (knockdown in HEK293T)

	gRNA
SIRT5	**GGCGCTCCGGACCTGAGCCA**
Non-targeting control	**GCTGCATGGGGCGCGAATCA**

Sequences from Horlbeck et al., 2016 [1]

Reference:

1. Horlbeck MA, Gilbert LA, Villalta JE, Adamson B, Pak RA, Chen Y, et al. Compact and highly active next-generation libraries for CRISPR-mediated gene repression and activation. Elife. 2016;5: e19760.

qPCR primers

	**Forward**	**Reverse**
SARS-CoV-2 gene N	**CACATTGGCACCCGCAATC**	**GAGGAACGAGAAGAGGCTTG**
OC43 gene N	**CCGACTAGGTTTCCGCCTGG**	**TCTGCTGGATGTGCGCGAAG**
GAPDH	**TTCTACAATGAGCTGCGTGTG**	**GGGGTGTTGAAGGTCTCAAA**
ACTIN	**CTGTTGCTGTAGCCAAATTCGT**	**ACCCACTCCTCCACCTTTGAC**
GFP/Nsp14	**AGCTAAGCTGGACAGCCAAT**	**ACCCGTCTTTGGATTAGGCA**
STAT3	**CTTTGAGACCGAGGTGTATCACC**	**GGTCAGCATGTTGTACCACAGG**
IL33	**GTGACGGTGTTGATGGTAAGAT**	**AGCTCCACAGAGTGTTCCTTG**
IFNB	**ATGACCAACAAGTGTCTCCTCC**	**GGAATCCAAGCAAGTTGTAGCTC**
IFITM2	**ATGAACCACATTGTGCAAACCT**	**CCCAGCATAGCCACTTCCT**
B2M	**TGTCTTTCAGCAAGGACTGGT**	**CTGCTTACATGTCTCGATCCCA**
SNCA	**AAGAGGGTGTTCTCTATGTAGGC**	**GCTCCTCCAACATTTGTCACTT**
IFIT3	**AAAAGCCCAACAACCCAGAAT**	**CGTATTGGTTATCAGGACTCAGC**

Antibodies

Strep-tag mouse	Qiagen #34850
Flag-tag rabbit	Cell Signaling #14793S
Beta-tubulin rabbit	Cell Signaling #2128S
SIRT1 rabbit	Cell Signaling #9475
SIRT2 rabbit	Cell Signaling #12650
SIRT3 rabbit	Cell Signaling #5490
SIRT5 rabbit	Cell Signaling 8779S
SIRT6 rabbit	Cell Signaling #12486
SIRT7 rabbit	Cell Signaling #5360
MAVS rabbit	Bethyl #A300-782A
Acetyllysine rabbit	PTM Biolab # PTM-105
Succinyllysine rabbit	PTM Biolab # PTM-401
Malonyllysine rabbit	PTM Biolab # PTM-901
Anti-mouse IgG, HRP-linked	Cell Signaling #7076
Anti-rabbit IgG, HRP-linked	Cell Signaling #7074

Small-molecule inhibitors and activators

Sirt5 inhibitor 1	Medchemexpress HY-112634
Sirt1 inhibitor Ex-527	Sigma-Aldrich E7034-5MG
NAMPT inhibitor FK866	Sigma-Aldrich F8557
Resveratrol	Sigma-Aldrich R5010-100MG
SRT1720	Selleckchem S1129
MG-132	Selleckchem S2619
Doxycycline	Sigma-Aldrich F9891
