# Supplementary material for: SIRT5 is a proviral factor that interacts with SARS-CoV-2 Nsp14 protein
Source: PLoS Pathog. 2022 Sep 12;18(9):e1010811. doi: 10.1371/journal.ppat.1010811 (PMC9499238; doi:10.1371/journal.ppat.1010811)
Supplement: Attachment — Submitted filename: PLOS rebutal.docx [file ppat.1010811.s007.docx]

Rebuttal letter

Dear editors,

Please find below our answer to reviewers' comments following our initial submission to *PLOS Pathogen*. We are thankful for the overall positive and constructive feedback provided by the reviewers. We have modified the text and added several figures that address the main concerns. We performed key experiments that clarified the role of SIRT5 during SARS-CoV-2 infection. In particular, we showed that SIRT5 role in immune activation is independent of the RIG-I/MAVS pathway, contrary to what could have been expected. This confirms the unexpected role of SIRT5 during viral infection and strengthens the interest in our work. While mechanistic details remain to be investigated, our work provides important insight into the biology of SARS-CoV-2, and highlights an unexpected function for SIRT5 as a proviral factor.

We are presenting a substantially improved version of the manuscript and believe that this revised manuscript is now suitable for publication. Please find below an answer to the editor’s decision letter, followed by a point-by-point answer to the reviewers’ comments.

Sincerely,

The authors.

*D*ecision letter and author response.

Dear Walter,

Thank you very much for submitting your manuscript "SIRT5 is a proviral factor that interacts with SARS-CoV-2 Nsp14 protein" for consideration at PLOS Pathogens. As with all papers reviewed by the journal, your manuscript was reviewed by members of the editorial board and by several independent reviewers. In light of the reviews (below this email), we would like to invite the resubmission of a significantly-revised version that takes into account the reviewers' comments.

While the overall consensus of reviewers was positive, they felt that some additional experiments are required to make this manuscript suitable for publication in PLOS Pathogens.

- We are thankful for the constructive feedback received from the reviewers and the opportunity to submit a revised manuscript. We have focused our efforts on answering the key points highlighted below.
- Major changes in the manuscript include:
  - In Fig 4. We have performed synergy experiments with SIRT5 and SIRT1 inhibitors, which show no further inhibition when both are used at the same time.
  - Fig 4 was broken down in two for clarity. The experiments with OC43 are now in Fig. 5
  - Fig 7 (old Fig 6) now includes confirmation by qPCR of the upregulation of innate immune markers in *SIRT5-*KO cells
  - Fig 8 includes an analysis of the effect of knocking out MAVS and inhibiting *SIRT5,* which importantly suggests that SIRT5 function is independent of MAVS signaling.
  - Sup Fig 1 includes an experimental assessment of the protein folding of *SIRT5* mutants

From the multiple experiments suggested by reviewers I am guiding the authors towards the following experiments for a revised manuscript:

1) To strengthen evidence for the proposed link between NSP14/SIRT5 and innate immune activation: This seems to be the major hurdle to overcome. I suggest following reviewer 1 suggestions in investigating in mechanistic detail the complex's action on factors of the innate immune cascade, i.e. MAVS. The authors should also examine whether expression of Nsp14 would induce changes in innate response similar to a loss or induction of SIRT5. The methodology of generating recombinant SARS-CoV-2 may not be open to the authors in order to follow through with modulating NSP14 binding to SIRT5 and probing for the impact on innate immune activation. As an alternative, the authors may utilize cellular tools to modulate innate activation, i.e. use of IFN-competent and -incompetent cells, or Jak/Stat inhibitors, and investigate whether the phenotype holds. Finally, SIRT5 KO cells complemented with a mutant of SIRT5 that lacks binding to Nsp14 could provide some insights.

- We agree with the reviewers and recognize that the mechanistic link between the Nsp14/SIRT5 interaction and the immune activation in *SIRT5-*KO is currently unclear. We performed several experiments to solve this issue, which brought interesting results and necessary clarifications.
- The most obvious potential link between SIRT5 and the immune response was through the RIG-1/MAVS pathway. Previous studies showed that MAVS is desuccynilated by SIRT5, which prevents MAVS aggregation and impairs interferon production. One leading hypothesis to explain the proviral role of SIRT5 was that SIRT5 prevented MAVS aggregation, in turn causing a weaker innate immune response. In Fig 8, we analyzed the effect of inhibiting SIRT5 in a MAVS knockdown. Interestingly, we found that SIRT5 was acting independently of MAVS, since inhibiting SIRT5 had the same effect in WT and MAVS-KD. This invalidated the hypothesis and showed that SIRT5 role in innate immunity is not limited to regulating MAVS. This result is important and significantly strengthens the interest in our manuscript. These results are included in Fig 8A-B.
- One major hurdle had been the impossibility of successfully analyzing *in vitro* the interaction between SIRT5 and Nsp14, which has prevented us from biochemically characterizing the complex. Following the reviewers' suggestions, we attempted to analyze in cells whether expressing Nsp14 would induce changes in innate responses similar to a loss or induction of SIRT5. One second hurdle is that Nsp14 is cytotoxic, which we had observed in transient transfection experiments in HEK293T. It was impossible to generate cells stably expressing Nsp14 using a lentiviral vector constitutively expressing Nsp14, as the vector killed the cells. We attempted to circumvent this toxicity issue by using a Doxycycline inducible lentiviral vector, but unfortunately, the cell lines we generated were not functional: While a GFP control could be strongly induced by Doxycycline, cells carrying inducible Nsp14 could not be induced. We think that leaky basal expression of Nsp14 was sufficient to negatively select against permissive integration events. We were disappointed not to be able to generate these cell lines, and due to time constraints, we could not further investigate the impact of Nsp14 on SIRT5 activity in cells. These results are presented in Fig 8C.
- Finally, we dedicated a lot of effort to complementation experiments as suggested, following, in particular, the suggestions of reviewer 3. As noted by reviewer 3: “*Since mutation Y102F maintains the binding to Nsp14, this could give a perfect tool to de-couple catalytic activity of SIRT5 from Nsp14 binding. If SIRT5 Y102F mutant can rescue the effect of SIRT5 KD on viral replication the authors could answer a key question if SIRT5-Nsp14 binding or just SIRT5 enzymatic activity is needed for proper viral replication*.”. We attempted to follow these suggestions and generated cell lines where SIRT5-KO was complemented by either WT SIRT5, SIRT5-H158Y, which is catalytically inactive and doesn’t interact with Nsp14, or SIRT5-Y102F, which is catalytically inactive but still interacts with Nsp14 (as shown in Fig 2B). These cell lines were built using a lentiviral vector expressing the different constructs or a GFP control under a constitutive EF1a promoter. lentiviruses were transduced at a low MOI to ensure that only around one vector copy got integrated per cell, but SIRT5 was nonetheless massively over-expressed in the three complemented cell lines compared to WT levels, more than 100x above WT level (Panel A below).
  These complemented cell lines were then infected with SARS-CoV-2 at MOI=0.5, and viral titers were measured after 3 days (Panel B below). As expected, SIRT5 could rescue the decrease in viral titers observed in *SIRT5-KO*, but SIRT5-H158Y could do so as well. This result was highly puzzling and hard to comprehend, as SIRT5-H158Y is catalytically inactive and does not interact with Nsp14. We repeated this experiment multiple times, and even re-genotyped the complemented cell line to ensure that samples had not been mixed along the way, but got consistent results.

After reflection, we decided not to include these results in the revised manuscript, as they make little sense and we are not confident that they are not an experimental artifact. We suspect that the supra-physiological amount of overexpressed protein has unintended consequences and might be causing artifacts that could explain these confusing results.
Repeating these experiments already forced us to ask for several extensions of the re-submission deadline, and we don’t have time to confirm or invalidate these results by other means. Besides, non-transduced WT and *SIRT5*-KO were propagated alongside the complemented cells, but had become poorly infectable by the time we performed these experiments (not shown). We repeatedly observe that A459-ACE2 cells that are being passaged for too long tend to lose their infectivity (probably because the ACE2 transgene is increasingly silenced), and this additional confounding factor was another reason not include this data.
Of note, we intend to keep investigating this puzzling observation by making new complemented cell lines that express a more physiological amount of SIRT5, but this will take us several months. If this result hold, which we doubt, it would indicate that a catalytically inactive SIRT5 has antiviral properties and that SIRT5 has additional functions besides its deacylated activity. This would go against everything that is known about SIRT5 and certainly warrant its own study.

- We were very disappointed not to be able to include these results, as it was one of the most interesting experiments suggested by the reviewers and we dedicated significant efforts to it. We were able to address almost all of the other reviewers’ comments (see above and below) and generated important new data that significantly strengthened the conclusion of our work. We hope that this omission will not prevent our manuscript to be accepted, as we truly gave our best effort.
  We acknowledge that the link between our two key findings (the Nsp14-SIRT5 interaction and the immune activation in SIRT5-KO) remains unclear despite our best efforts. However, and even if mechanistic details remain to be investigated, we believe that overall our findings are significant. The strong interaction between SIRT5 and Nsp14 is conserved in SARS-like coronaviruses and understanding its precise role during infection will be the subject of further investigation. Besides, showing that SIRT5 is a proviral factor constituted a surprising result that will be of interest in the field. SIRT5 is a key metabolic enzyme that regulates several important metabolic processes, but its role during disease and infection is currently unknown. Our results suggest that SIRT5, and potentially other sirtuins, could act as a bridge between cellular metabolism and the innate responses against viral infections. Our research opens a novel area of investigation and will be of special relevance for investigators interested in the crosstalk between metabolic pathways and antiviral responses.

2) To strengthen the physiological relevance of findings: Perform synergy experiments with combined SIRT1/5 inhibitors and/or SIRT5 inhibitors with remdesivir or other available antiviral drugs.

- We performed these experiments as suggested. Interestingly, treating A549-ACE2 or Calu3 cells with both SIRT5 and SIRT1 inhibitors did not further reduce viral levels. Titers significantly decreased by two to fourfold in cells treated with Sirt5-I and Ex-527 together, a level similar to cells treated with the inhibitors alone (Fig. 4F, H). This suggested that the roles of SIRT1 and SIRT5 during SARS-CoV-2 infection might be interdependent. These new results are presented and discussed in the manuscript.
- We performed experiments treating cells with both remdesivir and SIRT5/SIRT1 inhibitor as suggested. However, in our cell culture experiments, treating with remdesivir alone or in combination completely abrogated viral replication, (no plaques were counted in plaque assays). Remdesivir is so efficient by itself in cell culture that any additional effect of SIRT5/SIRT1 inhibition was impossible to measure. Data with Sirt5/Sirt1 inhibitor + remdesivir were therefore not included in the manuscript.
- The results of these experiments with single or multiple inhibitors reached high statistical significance with a high number of replicates (n=9), and we used this new data in the revised manuscript. In our original manuscript, we had high variation between experiments done on different days. In these new experiments, we were able to better control our experimental conditions to have very robust results, which makes our overall conclusion stronger. With this new data, the analysis performed in old Fig 4I that calculated a “plaque assay z-score” to account for experimental heterogeneity is now unnecessary and we removed it.

3) To strengthen mechanistic detail of SIRT5/NSP14 interaction: The path forward to biochemical studies seem to be barred by difficulties with protein purification. I suggest incorporating reviewer 3's comments by adding predicted 3D models of the different mutants, and otherwise tackling the reviewer's points in the in discussion section.

- To assess experimentally if SIRT5 mutants were folded properly, we measured protein stability in cells treated with the proteasome inhibitor MG-132, which offers a crude way to experimentally assess protein folding. We could not observe major differences with WT and concluded that protein folding was not dramatically affected. These results are included in Supplementary Figure 1.
- We attempted to use prediction tools to computational model the effect of the mutations. We tested different online tools, but results between the different tools gave widely different results, with the same mutation predicted to have either no effect or a strong destabilizing effect depending on the model used. These models are reported to be unreliable (PMID: 34225665) and we didn’t include this analysis.
- Additional considerations are addressed later in the rebuttal below reviewer 3 comments.

This editor recognizes that mouse experiments to perform suggested pathogenicity studies may be outside of the scope of this manuscript.

- We are thankful for this, as it would not have been possible for us to perform such studies as part of this revision process.

# Reviewer's Responses to Questions

## Part I - Summary

Please use this section to discuss strengths/weaknesses of study, novelty/significance, general execution and scholarship.

**Reviewer #1**: This manuscript by Walter, Verdin and colleagues examines the effects of the sirtuin SIRT5 in cellular models of SARS-CoV-2 infection. SIRT5 is a member of the sirtuin deacylase family; SIRT5 has been shown, in work by the Verdin group and others, to act as a cellular desuccinylase, demalonylase, and deglutarylase, rather than as a deacetylase. The authors find that SIRT5 interacts with Nsp14, a viral protein with multiple enzymatic activities, in a SIRT5-activity dependent manner. The authors further show that genetic or pharmacologic inhibition of SIRT5 results in modest decreases in viral mRNA levels and viral titer in cultured cells infected with SARS-CoV-2. Via transcriptomic studies, the authors show that SIRT5 impacts multiple pathways in cells, including autophagy and innate immunity.

It goes without saying that Covid-19 remains an enormous global public health challenge. Thus, identifying new players in SARS-CoV-2 pathobiology, such as SIRT5, is of great relevance. With that said, there are issues with this manuscript that need to be addressed before it is acceptable for publication, in my view.

- We thank reviewer 1 for their positive comments and did our best to answer their concerns and suggestions.

**Reviewer #2**: Viruses, including the pandemic SARS-CoV-2, need to manipulate the cellular environment to facilitate their own replication and propagation. To this end, they engage cellular factors and exploit them for their purposes. These pro-viral factors are often targeted and hijacked by viral proteins.

In this manuscript, Walter and colleagues identified the cellular deacylase/desuccinylase/demalonylase SIRT5 (and SIRT1) as an interaction partner of the SARS-CoV-2 non-structural protein 14. While the interaction depends on the activity/active domain of SIRT5, it does not remove PTMs from Nsp14. The authors propose that by targeting SIRT5, SARS-CoV-2 reduces innate immune activation, as cells lacking SIRT5 show increased levels of known ISGs that antagonise SARS-CoV-2.

Overall the data presented in the manuscript is already quite convincing, generally supports the main conclusion and certainly provides an interesting and novel description of a (non-methylase) function of Nsp14 and identification of its cellular interaction partner SIRT5 as a pro-viral protein. However, the link between Nsp14/SIRT5 and the innate immune induction phenotype is a bit weak, as well as the impact of the Nsp14-SIRT5 interaction on virus replication.

I have only a few suggestions to improve the current manuscript.

- We thank reviewer 2 for their positive appreciation of our manuscript!

**Reviewer #3**: Walter et al. demonstrates that SIRT5 (and to some extent SIRT1) facilitates SARS-CoV-2 replication in vitro. Both proteins stably interact with Nsp14, and the catalytic activity of SIRT5 and SIRT1 are necessary for this interaction. Based on their results, they propose SIRT5 and SIRT1 to be proviral factors and could be a new therapeutic targets for Covid19 treatment.

The authors show compelling evidence that SIRT5 binds Nsp14 using immunoprecipitations from cell lines. They also prove that SIRT5 catalytic activity is needed for this interaction and that SIRT5/SIRT1 KD or inhibition lowers viral propagation in cell lines. Experiments are performed at high quality and the manuscript is well written, however the manuscript presented here has some key limitations.

- We thank reviewer 3 for their positive comments and did our best to answer their concerns and suggestions.

## Part II – Major Issues: Key Experiments Required for Acceptance

Please use this section to detail the key new experiments or modifications of existing experiments that should be absolutely required to validate study conclusions.

Generally, there should be no more than 3 such required experiments or major modifications for a "Major Revision" recommendation. If more than 3 experiments are necessary to validate the study conclusions, then you are encouraged to recommend "Reject".

**Reviewer #1**: 1. The authors show that SIRT5 interacts with Nsp14, but also that SIRT5-inhibited cells show an enhanced innate immune activation state that actually preexists infection. Do the authors have any evidence that the SIRT5-Nsp14 interaction actually has any role in modulating the outcome of SARS-CoV-2 infection? It seems to me that this interaction may be irrelevant to the role of SIRT5 in promoting viral pathogenesis identified by the authors, which could be driven exclusively by the effects of SIRT5 genotype on innate immunity.

- These concerns were addressed in more detail in the first paragraphs of this rebuttal. We agree with reviewer 1 that the potential link between the Nsp14/SIRT5 interaction and the immune phenotype is the key question remaining in our manuscript. It is indeed possible that the two observations are independent of each other, and our results show that they are at least partially independent. As described above, we attempted to investigate whether overexpression of Nsp14 alone could have an impact on SIRT5 activity, but these experiments were unsuccessful because of the high cytotoxicity of Nsp14.

2. The effects of SIRT5 deficiency/inhibition on viral titers seem quite modest, mostly less than a log-fold. Thus, the physiologic relevance of these findings remains somewhat uncertain. Since the authors also identify a role for SIRT1 inhibition in modulating cellular SARS-CoV-2 responses, perhaps it is worth testing the effects of combined SIRT1/SIRT5 inhibition, or the effects of combing SIRT5 inhibition together with known effective anti-viral therapy (e.g. remdesivir, Paxlovid, and the like).

- Indeed we were careful in the manuscript not to exaggerate the effect of SIRT5 knockout or inhibition, as the overall effect is modest, in the order of 1-log, as noted by this reviewer. We however believe that these results are meaningful, as they highlight an unexpected role for SIRT5 during SARS-CoV-2 infection.
- We performed the requested experiments and investigated the effect of combined SIRT1/SIRT5 inhibition. Interestingly, we didn’t observe any synergistic effect. Treating A549 or Calu3 cells with inhibitors together was not different than treating cells with inhibitors alone. This suggests that SIRT5 and SIRT1 roles might be interdependent or act in the same pathway. These new results are presented and discussed in the manuscript (Fig 4).
- Remdesivir alone can almost completely abrogate viral replication in cell culture, and we could not measure an additional effect of SIRT5/SIRT1 inhibition, as discussed above.

3. The manuscript would be much stronger if the authors had evaluated SIRT5 KO mice for their response to SARS-CoV-2 infection, and this might address point 2. Such animals might well show improved responses over those observed in cell culture, since the totality of their immune responses might well be enhanced (not only through cell-autonomous effects in the cells themselves). I fully recognize the logistical difficulties involved in such studies, which may be outside the scope of this manuscript.

- We agree that a mouse model of SARS-CoV-2 infection in SIRT5-KO mice would be exciting, and we hope to be able to perform such studies in the future. We are thankful that the editors recognized that these experiments are out of the scope of the present manuscript, because of logistical, funding, and time constraints.

**Reviewer #2**: Major issues:

While the authors check in detail whether SIRT5 regulates PTMs on Nsp14, and find no evidence of that they should check whether binding of Nsp14 to SIRT15 alters its impact on other cellular factors, especially in the IFN cascade, e.g. MAVS (K7 succinylation). Furthermore, the authors should examine whether expression of Nsp14 would induce changes in innate response similar to a loss or induction of SIRT5. This may provide a link between Nsp14 and innate immune defences. Ideally, a recombinant SARS-CoV-2 virus lacking binding of Nsp14 to SIRT5 would be more immunostimulatory and attenuated in immunocompetent cells.

- We thank reviewer #2 for their suggestions and we attempted to address them as best as we could.
- As explained in detail above, we investigated if SIRT5 was acting through the MAVS pathway, and showed that SIRT5 role was likely independent of MAVS during SARS-CoV-2 infection, which represented a key result.
- We also attempted to investigate whether the expression of Nsp14 would induce changes in innate immunity similar to the loss of SIRT5. However, Nsp14 is cytotoxic and we could not generate cell lines that stably overexpress Nsp14, using either constitutive or inducible expression vectors. We were very disappointed not to be able to generate these cell lines, as they would have represented a key tool to investigate the role of the Nsp14 in modulating SIRT5 activity.
- As noted by the editors, generating recombinant SARS-CoV-2 was not a technology that was easily available to us. Besides, while we showed that a functional catalytic domain of SIRT5 is necessary for the interaction with Nsp14, we currently don’t know which domains of Nsp14 are involved in the interaction. We are planning in the future to generate mutants of Nsp14 and analyze their interaction with SIRT5. However, finding Nsp14 mutants that lack SIRT5 binding but retain functional MTAse and proofreading activity will require extensive characterization that is out of the scope and logistical constraints of this revision process.

Alternatively, SIRT5 KO cells complemented with a mutant of SIRT5 that lacks binding to Nsp14 could provide some insights (ideally with intact catalytic activity!).

- Complementing cells with a SIRT5 mutant that lacks binding to Nsp14 but with intact catalytic activity is an attractive idea that is however likely unfeasible. We showed with multiple assays that SIRT5 activity is necessary for the interaction, and all the SIRT5 mutants we investigated that lost the interaction were also catalytically inactive (Fig 2B).

It is a bit unusual that catalytic activity necessary for binding. As the in vitro assays suggest it is a direct binding, can this be outcompeted in vitro by a SIRT5 inhibitor?

- We agree that the finding that the catalytic activity is necessary is unusual, and our first hypothesis was that SIRT5 was directly modifying Nsp14. However our mass spectrometry experiments invalidated this hypothesis, and we could not find any evidence that Nsp14 was directly deacylated. We discussed this observation at length in the discussion.
- We showed in Fig 2C that treating cells with a SIRT5 inhibitor suppressed the binding, which answers the last question.

**Reviewer #3**: Key limitations:

(i) The authors were not able to reconstitute the SIRT5-Nsp14 complex in vitro using recombinant proteins purified from E. coli. This means that the authors are probably missing key components of the complex as these two proteins might not be binding each other directly. This would be a key mechanistic point to try to tackle (for example with mass spectrometry identification of SIRT5-Nsp14 complex components).

- We agree that this is a limitation of our study. Two other high-confidence interaction partners of Nsp14 have been identified by Mass Spectrometry (GLA and IMDH2), and we confirmed them in our own Mass spectrometry. It will be interesting in the future to investigate whether SIRT5, IMDH2 and GLA are interacting together.

(ii) The authors show that Nsp14 from HCoV-OC43 does not bind SIRT5. They also show that SIRT5 catalytic inhibition also lowers HCoV-OC43 replication efficiency. This suggests that SIRT5 might be exerting its antiviral properties completely independently from binding Nsp14.

- We slightly disagree with this interpretation of our results. The experiment with OC43 indicates that SIRT5 is exerting its antiviral activity that is at least partially independent from binding Nsp14. It is possible indeed that the effect is completely independent, but our experiments do not allow us to conclude that. Since the catalytic activity of SIRT5 is necessary for the interaction, it is difficult to untangle the role of the interaction versus other roles of SIRT5. The key experiments to address this point would be to generate recombinant SARS-CoV-2 virus that lacks SIRT5 binding but retain a functional Nsp14, as suggested by reviewer 2. However as discussed above, this type of analysis is currently out of reach until we can characterize the interaction in further detail.
- To better highlight this important comment of reviewer 3, we have moved the experiments with OC43 into a dedicated figure (new Fig 5)

Please see my specific comments below:

Major points:

1.) Figure 2B shows the binding between Nsp14 and SIRT5 catalytic mutants. Some mutations completely abolish binding while others have no or only mild effect. It is not evident if the mutations (and the Sirt5-i) affect the folding of SIRT5 and that causes the reduction in binding and enzymatic activity. The authors should establish experimentally the effect of these mutations on SIRT5 enzymatic activity and the folding of the protein.

- To assess experimentally if SIRT5 mutants were folded properly, we measured protein stability in cells treated with the proteasome inhibitor MG-132, which offers a crude way to determine protein folding experimentally. We could not observe significant differences with WT and concluded that protein folding was not dramatically affected. These results are included in Supplementary Figure 1.
- As discussed above, we also attempted to use computational tools, but the results were inconsistent between different tools and we didn’t include this analysis.

Since mutation Y102F maintains the binding to Nsp14, this could give a perfect tool to de-couple catalytic activity of SIRT5 from Nsp14 binding. If SIRT5 Y102F mutant can rescue the effect of SIRT5 KD on viral replication the authors could answer a key question if SIRT5-Nsp14 binding or just SIRT5 enzymatic activity is needed for proper viral replication.

- This was a great suggestion and a clever experiment. As discussed at length above, we dedicated significant effort to it but decided against including the results. We are not confident that they are not experimental artifacts, and if they hold true, would require more extensive investigation.

2.) In Figure 3D and E the authors claim that SRT1720 and Resveratrol do not have an effect on Nsp14 and SIRT1 binding. Based on their blots, there is a clear reduction in binding between Nsp14 and SIRT1 when SRT1720 is used at 25, 50 uM and Resveratrol used at 25 and 100 uM.

- This reviewer is correct and there is an apparent reduction in binding with SRT1720 and Resveratrol. However, a confounding factor is that the overall expression level of SIRT5 and Nsp14 is also reduced with high concentrations of the activators, which is observable in the “input” lanes of Fig 3D and E. The apparent decrease in binding, therefore, correlates exactly with a decrease in expression. In these experiments, we observed that high levels of the two activators were really cytotoxic, which probably explains the decrease in expression.
- We modified the text to better illustrate this interpretation and address this reviewer's comment.

3.) Unfortunately, the authors were not able to uncover the molecular functions of the Nsp14/SIRT5 complex. In Figure 3G the authors only show the effect of SIRT5 on the methyltransferase activity of Nsp14 (does not affect it). They state that since Nsp10 is needed for the ExoN activity of Nsp14 and that SIRT5 and Nsp10 are in separate complexes they did not evaluate the potential effect of SIRT5 on the ExoN activity of Nsp14. While this is true, the authors should have considered the following hypothesis: They have proved that Nsp10 and SIRT5 is competing to bind Nsp14. Therefore, SIRT5 might inhibit Nsp14 ExoN activity during viral replication by outcompeting Nsp10, which has been shown to be vital for SARS-CoV-2 replication.

- This is a good point and we are thankful that the reviewer agrees with our overall interpretation. The hypothesis suggested by this reviewer is interesting but would not easily fit with the rest of our data. If SIRT5 was inhibiting Nsp14 ExoN activity by outcompeting Nsp10, one might expect that knocking out SIRT5 would result in increased ExoN activity and potentially higher viral levels. We, however, observed the opposite and SARS-CoV-2 levels were decreased without SIRT5. Besides, by analyzing RNA-seq reads, we could not measure any difference in the mutation levels of SARS-Cov-2 RNA in the SIRT5-KO, which would have been expected if SIRT5 was implicated in the ExoN activity. It is possible that SIRT5 is involved in fine-tuning the Nsp14/Nsp10 interaction, but the effect is likely limited.
- In our discussion and in the different models presented in Fig 8, we proposed an alternative hypothesis that fits better with the rest of our data. We propose that SIRT5 and Nsp10 could be separately involved in the two enzymatic activities of Nsp14, with the Nsp14/SIRT5 complex primarily responsible for viral mRNA Cap methylation (model 3).

## Part III – Minor Issues: Editorial and Data Presentation Modifications

Please use this section for editorial suggestions as well as relatively minor modifications of existing data that would enhance clarity.

**Reviewer #1**: 4. (Minor): in their Nsp14 IP/IBs, the authors should examine lysine glutarylation, in addition to the marks examined.

- This is a good point. We didn’t include that in our western blot analysis, but glutarylation was examined by mass spectroscopy in Fig 2F and we didn’t detect any changes either. It was already included in the text, but we modified Figure 2F to clarify this point.

**Reviewer #2**: Minor issues:

A double KO of SIRT1 or SIRT5 should be added, to show whether there are two independent functions of these protein targeted, or whether it is additive effects.

- We have added experiments conducted with two inhibitors. This is not as robust as the experiment suggested with knockouts, but was more amenable to the review process. As discussed above, we didn’t observe any additive effects, suggesting that the two proteins act interdependently.

I strongly recommend to improve the figure legends, i.e. show number of repeats, statistics used, antibodies used etc

- The numbers of biological replicates and statistics are already indicated in legends. The antibodies used are available in the supplementary tables at the end of the method section.

The western blots would benefit from black boxes around them, currently the fade into the white paper background too much as the contrast is very high.

- We attempted to carry on this modification, but, in our opinion, it made the figures too cluttered and less readable, so we decided against following this suggestion.

The phrase ‘stably interact’ sounds a bit odd to me and is used multiple times throughput the manuscript. What do the authors mean by this?

- We removed “stably”, as we agree that this is redundant.

Fig. 1 C: Please add a quantification of the colocalization

Positive controls for e.g. a SIRT5-dependent deacetylation missing in Fig. 2E.

- This is a good point and additional controls would have made the results more compelling. Fortunately, the more rigorous mass spectrometry analysis of Fig 2F makes the western blots of Fig 2E redundant. We included the westerns because they provide visual results and we believe they are still informative, but we concentrated our effort on the mass spectrometry analysis.

Fig. 2F: Can the authors state whether putative PTM sites were covered by the peptides in the MS?

- We had previously stated that our MS analysis had close to 90% coverage, and further refined this number by calculating that 94% of lysines in Nsp14 were covered. Only 2 out of 30 lysines were not covered. It is possible that one of these two lysines was actually acylated, but we also didn’t see any changes by western blot. We modified the text of the results and discussion to include this refined coverage number.

Fig. 3A: Please include SIRT5 as a control and show the IP blot of Strep.

- The controls for Fig 3A are actually in Fig 1B, since these experiments were performed with the same samples. This is explained in the figure legend

**Reviewer #3**: Minor points:

1.) Figure 5 shows the up and down regulated genes in the SIRT5 WT vs. KO cells w/wo viral infection. The authors should discuss further how many of the genes with differential expression have the potential to restrict viral replication in an in vitro environment without a functional innate immune system present in their assays.

- We included in Fig 7B a validation by qPCR of the RNA-seq results, which strengthened our results.
- While this reviewer’s suggestion is a good one in principle, we feel that providing a definite number of the genes with the potential to restrict viral replication in vitro would be very artificial. The genes that we included have been annotated in curated databases for genes in immunity pathways. Following this suggestion rigorously would mean validating experimentally for our specific cell type each of these genes individually, which is not reasonably feasible considering that there are hundreds of genes included in these pathways.
